# The effect of de-escalation simulation training on empowerment and confidence in managing patient aggression among psychiatric nursing students: an experiential learning approach

**DOI:** 10.1186/s12912-025-03958-1

**Published:** 2026-01-12

**Authors:** Gamal Ali Abdelhamid Eltrass, Sorayia Ramadan Abdelfattah, Ghada Mohammed Mourad, Mostafa Shaban, Wafaa Osman Abd El-Fatah

**Affiliations:** 1 Department of Psychiatric and Mental Health Nursing, Faculty of Nursing, October 6 University, Giza, Egypt; 2 Department of Psychiatric and Mental Health Nursing, Faculty of Nursing, Ain Shams University, Cairo, Egypt; 3 Geriatric Nursing Department, Faculty of Nursing, Cairo University, Cairo, Egypt; 4 Present Address: Professor of Psychiatric Mental Health Nursing, Faculty of Nursing, Helwan University, Cairo, Egypt

**Keywords:** Psychiatric nursing, De-escalation, Simulation training, Clinical confidence, Psychological empowerment, Nursing education, Experiential learning

## Abstract

**Background:**

Patient aggression is a persistent challenge in mental health settings, and undergraduate preparation in de-escalation remains variable. This study evaluated whether a standardized-patient (SP) de-escalation simulation improves psychological empowerment and confidence in coping with aggression among psychiatric nursing students.

**Methods:**

We conducted a parallel-group randomized controlled trial (pretest–posttest) with undergraduate psychiatric nursing students randomly assigned to either an SP-based de-escalation simulation program or routine curriculum control. The intervention comprised 12 sessions over 8 weeks (4 theory + 8 SP practical scenarios with structured debriefs aligned to INACSL standards). Primary outcomes were psychological empowerment and confidence in coping with patient aggression, assessed at baseline and immediately post-intervention using validated scales. Fidelity was supported through scripted scenarios, SP calibration, and facilitator adherence/debrief quality checks. Between-group differences in change scores were examined.

**Results:**

Compared with the control group, students in the simulation arm demonstrated significantly greater gains in both psychological empowerment and confidence in coping with aggression from pretest to posttest. Fidelity indices indicated consistent SP portrayal and facilitator adherence across sessions, and attendance was high. No adverse events were reported.

**Conclusions:**

A structured de-escalation simulation using trained standardized patients significantly improves empowerment and confidence among psychiatric nursing students. The scenario maps, learning outcomes, and debriefing approach described support replication and curriculum integration in undergraduate mental health nursing. Future work should examine longer-term retention, transfer to clinical practice, and curriculum-level outcomes.

**Clinical trial number:**

Not applicable.

**Supplementary Information:**

The online version contains supplementary material available at 10.1186/s12912-025-03958-1.

## Introduction

In contemporary mental health care environments, the incidence of patient aggression remains a persistent and growing concern, especially in psychiatric settings. Nursing students, as novice practitioners, often find themselves unprepared to manage violent or aggressive behaviors, placing both their safety and the therapeutic environment at risk [1]. Workplace violence (WPV), encompassing verbal abuse, physical assault, and psychological threats, has become an alarming global issue in nursing education and practice [2]. Reports indicate that nursing students are among the most vulnerable healthcare workers exposed to WPV due to limited experience, lack of confidence, and insufficient training in handling high-stress interpersonal scenarios [2, 3].

The psychiatric clinical setting presents unique challenges for undergraduate nursing students. These include managing complex symptomatology such as paranoia, hallucinations, and unpredictable behavioral outbursts, often underpinned by patients’ past trauma or co-occurring substance use disorders [4]. While clinical exposure is fundamental to developing therapeutic skills and professional identity, the unpredictability of mental illness can generate significant anxiety and fear among students, thereby impeding learning and diminishing confidence [5, 6]. Negative first encounters with aggression or a lack of adequate coping strategies may also dissuade students from pursuing a future in psychiatric nursing [7].

A critical concern in psychiatric nursing education is the growing gap between theoretical instruction and real-world practice, particularly in the domain of aggression management. Traditional pedagogical approaches often fail to address the affective and behavioral responses necessary to engage with agitated or aggressive patients effectively [8]. Clinical rotations, though valuable, are increasingly constrained due to rising concerns around patient safety, limited clinical sites, and legal restrictions on student-patient interaction in psychiatric settings [9]. Consequently, there is a pressing need for innovative educational strategies that prepare students not only with knowledge, but also with the practical skills and confidence to manage aggression safely and empathetically [10].

Simulation-based education has emerged as a transformative tool in nursing pedagogy, offering students a structured and safe environment to practice clinical decision-making and interpersonal communication. High-fidelity simulation using standardized patients (SPs) allows learners to engage in realistic scenarios, receive immediate feedback, and reflect critically on their responses to challenging situations [11]. These immersive experiences are particularly valuable in psychiatric nursing, where therapeutic communication and emotional regulation are paramount. Simulated encounters with agitated patients can serve as an effective proxy for real-life aggression exposure, enabling students to develop and refine de-escalation techniques without jeopardizing safety [12].

De-escalation is defined as a series of psychosocial and communicative strategies aimed at preventing or mitigating the escalation of aggressive behavior [13]. It encompasses verbal and non-verbal approaches such as active listening, non-threatening posture, empathetic engagement, and boundary-setting skills that require practice, emotional intelligence, and situational awareness [14]. Despite the clear relevance of de-escalation in psychiatric care, formal training in these techniques is often absent from undergraduate nursing curricula. When such training is provided, it tends to be didactic rather than experiential, limiting the development of practical competence [15].

Research suggests that simulation-based de-escalation training not only improves students’ clinical competence but also significantly enhances psychological empowerment—a construct reflecting one’s perceived ability to influence their work and environment [16]. Empowerment is particularly important in nursing education, as it fosters autonomy, confidence, accountability, and professional resilience [17]. Psychological empowerment comprises four key dimensions: meaning, competence, self-determination, and impact [18]. When students feel empowered, they are more likely to take initiative, engage meaningfully in clinical learning, and demonstrate leadership in patient care [19].

Closely related to empowerment is clinical confidence, particularly in high-stress situations such as responding to aggression. Confidence in coping with patient aggression involves a sense of preparedness, perceived safety, and belief in one’s communicative and physical intervention abilities [20]. Studies have shown that simulation experiences enhance these affective attributes, making students more likely to act effectively and with composure during clinical encounters [21]. Enhancing both empowerment and confidence may ultimately lead to improved patient outcomes, reduced incidents of restraint or seclusion, and a safer therapeutic milieu [22].

## Aim of the study

The aim of this study was to evaluate the effect of de-escalation standardized patient simulation training on psychological empowerment and confidence in coping with patient aggression among university psychiatric nursing students.

### Research question

To what extent does de-escalation standardized patient simulation training influence psychological empowerment and confidence in coping with patient aggression among university psychiatric nursing students?

### Hypotheses

De-escalation standardized patient simulation training will significantly improve psychological empowerment and confidence in coping with patient aggression among university psychiatric nursing students.

## Methods

### Study design

We employed a parallel-group randomized controlled trial with pretest–posttest assessments to evaluate the effect of a standardized-patient de-escalation training on students’ psychological empowerment and confidence in coping with patient aggression. A computer-generated random sequence was prepared by an independent faculty member; allocation was concealed using sequentially numbered, opaque, sealed envelopes opened at the point of assignment. Outcome assessors and data analysts were independent of the teaching team. The control arm received the routine curriculum without simulation; the intervention arm received the de-escalation simulation program detailed below.

### Study setting

The study was conducted at the Psychiatric and Mental Health Nursing Department, Faculty of Nursing, October 6 University, located in Giza Governorate, Egypt. The Faculty of Nursing provides comprehensive education programs and is equipped with modern simulation labs specifically designed to train undergraduate nursing students in various clinical skills, including psychiatric nursing interventions.

### Sample and sampling

Undergraduate psychiatric nursing students enrolled in the mental health course during the study semester were invited to participate. Eligibility required concurrent enrollment in the course and consent to complete all study assessments; students with prior formal de-escalation training beyond routine coursework were excluded. All participants provided written informed consent.

Participants were allocated 1:1 to the experimental (de-escalation simulation) or control (routine curriculum) arm using a computer-generated random sequence prepared by an independent faculty member not involved in teaching, assessment, or data analysis. Allocation concealment was ensured with sequentially numbered, opaque, sealed envelopes (SNOSE) prepared off-site; envelopes were opened only after baseline measures were completed to prevent foreknowledge of assignment. Group sizes were balanced by the random sequence; no changes to allocation occurred post-randomization.

To reduce contamination, experimental and control sessions were scheduled separately, and students were asked not to share instructional materials or details of simulated scenarios across groups. Outcome assessors and data analysts were independent of the teaching team and blinded to group assignment. Attendance was recorded at each session to document the exposure dose.

### Control group

Students in the control arm completed the standard psychiatric nursing course content without simulation or role-play. The curriculum comprised 12 contact hours divided into three thematic blocks:

- **Therapeutic Communication (4 h)**: Covered establishing rapport, empathy versus sympathy, active listening, use of open/closed questions, non-verbal communication, and setting professional boundaries.
- **Crisis Intervention (4 h)**: Addressed crisis types, safety planning, triage, staff roles, and referral pathways.
- **Aggression Management Theory (4 h)**: Focused on recognizing risk factors, identifying early warning signs, reviewing organizational policies, and outlining safe team responses.

Learning outcomes for the control group emphasized cognitive knowledge (describe warning signs, outline de-escalation principles, identify staff roles) rather than skills practice. Teaching methods included lectures with PowerPoint, guided readings, and facilitated classroom discussions. Assessment consisted of short quizzes and formative in-class Q&A. Importantly, no standardized patients, scenarios, or role-play exercises were used in the control condition. *(See Supplementary Table *S1* for a session-by-session curriculum map with topics*,* objectives*,* readings*,* and in-class activities.)*

### Intervention (Simulation Scenarios)

Students in the experimental arm received a structured de-escalation simulation program using trained standardized patients (SPs) across 12 sessions over 8 weeks (4 theory, 8 practical).

Teaching and practice approach: Each practical session followed the sequence briefing → scenario (10–15 min) → coached deliberate practice on micro-skills → structured debrief. Scenarios were scripted to reflect realistic psychiatric ward encounters.

### Core micro-skills taught and reinforced included

1. Active listening and minimal encouragers.
2. Emotion labeling and validation.
3. Calm stance, posture, and safe distancing.
4. Clear limits and offering choices.
5. Alternative offers (quiet room, time-out, basic needs).
6. Supportive language for refusals.
7. Team signaling and escalation procedures.
8. Closure and recovery (summarizing, safety check, documentation).

Learning outcomes for each scenario were mapped to specific micro-skills, enabling students to demonstrate applied competence in early recognition, verbal de-escalation, and safe response strategies.

Debriefing followed the PEARLS blended approach, with faculty guiding reflection, eliciting cognitive frames, and linking student actions to patient safety.

Fidelity measures included use of scripted cue cards, props, timing windows, and SP calibration workshops. SPs were blinded to group allocation and to study hypotheses to minimize bias. Faculty completed scenario adherence checklists to ensure delivery consistency.

*(See Supplementary Table *S2* for a scenario map with session objectives*,* targeted micro-skills*,* fidelity elements*,* and debrief prompts.)*

### Data collection tools

Three data collection instruments were utilized:

#### Socio-demographic characteristics sheet

This tool was developed by the researcher following an extensive review of relevant literature. It aimed to gather demographic and background information such as gender, nationality, cumulative grade point average (CGPA), previous experience with simulation labs, prior experience with patient aggression, and fear related to psychiatric patient interactions.

#### Psychological Empowerment Scale (PES)

The Psychological Empowerment Scale (PES), originally developed by Spreitzer (1995) and later adapted and validated by Ruiz-Fernández et al. (2022), was employed to measure psychological empowerment among nursing students [23, 24]. This scale consists of 12 items divided equally into four dimensions: meaning (3 items), competence (3 items), self-determination (3 items), and impact (3 items). Each item is scored using a 7-point Likert scale ranging from (1) “completely disagree” to (7) “completely agree.” Total scores range from 12 to 84, with higher scores indicating greater psychological empowerment. The scoring categories are interpreted as follows: <50% indicating poor empowerment, 50–74% moderate empowerment, and ≥ 75% high empowerment. The scale’s validity and reliability were previously established, demonstrating strong internal consistency (Cronbach’s alpha >0.90). For the purpose of the current study, the PES was translated into Arabic and validated through a rigorous translation-back translation process, followed by expert panel review involving five experts in psychiatric nursing education from Egyptian universities. Minor linguistic and cultural adjustments were made based on expert recommendations.

#### Clinical confidence in coping with patient aggression questionnaire (CCWPA)

The Clinical Confidence in Coping with Patient Aggression questionnaire, originally developed by Thackrey (1987) and subsequently adapted by Kruse (2021), aimed to assess nursing students’ perceived self-confidence in coping with patient aggression [25, 26]. This questionnaire consists of 10 items rated on an 11-point Likert scale, where item responses range from negative perceptions such as “very uncomfortable,” “very poor,” “very unable,” “very unsafe,” and “very ineffective,” to positive perceptions including “very comfortable,” “very good,” “very able,” “very safe,” and “very effective.” Total possible scores range from 10 to 110, with higher scores indicating greater clinical confidence. Scores were categorized into three groups: <50% (low confidence), 50–74% (moderate confidence), and ≥ 75% (high confidence). The reliability and validity of this instrument have been previously established with strong internal consistency and criterion-related validity. Similar to the PES, the CCWPA was translated into Arabic, validated by a panel of experts, and minor cultural modifications were made to ensure appropriateness for the study population.

### Data collection procedure

Data collection was carried out in three main phases during the Fall semester of the academic year 2023–2024:

- **Preparatory Phase**: Permission to conduct the study was obtained from relevant authorities, and detailed orientation was provided to all participants. Baseline data (pre-training) were collected from both experimental and control groups using the structured electronic self-reported questionnaire, including demographic details, PES, and CCWPA questionnaires. Completion of the questionnaires took approximately 20 min.
- **Implementation Phase**: The experimental group received the de-escalation standardized patient simulation training over eight weeks, consisting of 12 structured sessions (4 theoretical and 8 practical). The sessions incorporated comprehensive scenarios and debriefing practices adhering to the standards of the International Nursing Association for Clinical Simulation and Learning (INACSL). Conversely, the control group continued their regular curriculum and received standard educational methods.
- **Evaluation Phase**: Immediately after the training, post-intervention data were collected from both groups using the same tools. The researcher monitored data collection closely to ensure accuracy and completeness.

### Fidelity and standardization

To ensure intervention fidelity and minimize bias, several procedures were implemented. Scenario fidelity was maintained through standardized scripts, cue cards, and an environment/props checklist to ensure consistent conditions across sessions. Timing windows were set at 10–15 min per scenario (± 2 min), with facilitators instructed to stop or redirect if scenarios exceeded this frame.

Standardized patient (SP) preparation: SPs underwent a 6-hour training workshop including calibration rehearsals, portrayal coaching, and consistency checks using a Scenario Portrayal Checklist. SP performance was reviewed periodically by faculty observers to ensure uniform delivery.

Blinding: SPs were not informed of group allocation and were scheduled with neutral session labels to reduce bias. While complete blinding could not be guaranteed due to logistical factors (separate scheduling of groups), SPs were blinded to study hypotheses and instructed to deliver scripted behaviors identically for all encounters.

Faculty adherence: Instructors followed a Scenario Adherence Checklist outlining learning objectives, sequence, and debriefing procedures. Approximately 20% of sessions were co-rated by a second faculty observer to establish inter-rater agreement, which consistently exceeded 85%. Attendance was recorded at each session to document exposure dose.

### Debriefing approach and quality assurance

Debriefing was conducted according to the International Nursing Association for Clinical Simulation and Learning (INACSL) Standards. Each session followed the PEARLS blended approach (Promoting Excellence and Reflective Learning in Simulation), which integrates descriptive reflection, analysis using advocacy–inquiry, and application to future practice.

Facilitator competencies: Faculty were trained in establishing psychological safety, eliciting learners’ cognitive frames, and linking observed behaviors to patient safety and professional practice. Facilitators used open-ended prompts, guided reflection, and feedback anchored in observed micro-skills.

Quality assurance: To monitor and standardize debriefing quality, facilitators completed the Debriefing Assessment for Simulation in Healthcare—Student Version (DASH-SV) after each session. Peer spot-checks were also conducted periodically to promote consistency. This structured approach ensured that debriefings were learner-centered, reflective, and aligned with session objectives.

### Data analysis

Data analysis was conducted using the Statistical Package for Social Sciences (SPSS), version 22.0. Descriptive statistics (frequencies, percentages, means, and standard deviations) were employed to summarize demographic and study variables. Comparisons of quantitative data between groups were made using Student’s t-test for normally distributed data and Mann-Whitney tests for non-normally distributed variables. Chi-square tests assessed categorical variables, and Spearman correlation coefficients evaluated relationships between psychological empowerment and clinical confidence levels. The significance level was set at *p* < 0.05.

### Ethical considerations

Ethical approval was obtained from the Scientific Research Ethics Committee, Faculty of Nursing, Helwan University (IRB No. HNUR-2023-09; approval date: September 15, 2023). Participants were informed about the study’s aim, procedures, and voluntary nature. Informed consent was signed by all students before participation, clearly stating the right to withdraw at any stage without consequence. Confidentiality and anonymity were assured, and data were securely managed. Ethical principles, including respect for autonomy, privacy, and cultural sensitivities, were strictly adhered to throughout the research process. This study was conducted in accordance with the ethical standards of the institutional research committee and with the 1964 Helsinki Declaration and its later amendments or comparable ethical standards.

## Results

The results section presents data gathered from 80 psychiatric nursing students, distributed equally into experimental (*n* = 40) and control (*n* = 40) groups.

### Socio-demographic characteristics of participants

Table 1 presents the socio-demographic characteristics of the nursing students in both the experimental and control groups. A total of 80 psychiatric nursing students participated in the study, equally distributed into experimental (*n* = 40) and control (*n* = 40) groups. The mean age of participants was 20.8 ± 1.2 years, with an overall range of 19–23 years, and no significant difference between groups.

In terms of gender, males comprised 62.7% of the experimental group and 65.2% of the control group. Regarding academic performance, slightly more than half of the experimental group (54.8%) and fewer in the control group (47.3%) had a cumulative GPA ≥ 2.0. Nationality was predominantly Egyptian in both groups, with one non-Egyptian student (2.3%) in the control group.

Concerning clinical exposure, 32.5% of the total sample reported prior experience with psychiatric clinical training or rotations, and 40.0% reported previous direct exposure to patient aggression. Additionally, 55.0% of students indicated fear or apprehension in dealing with psychiatric patients prior to the intervention.

**Table 1:** Socio-demographic characteristics of participants (*n* = 80)

Characteristics	Experimental group (*n* = 40) N (%)	Control group (*n* = 40) N (%)	χ²	*P*-value
**Gender**			0.06	0.816
Male	25 (62.7%)	26 (65.2%)		
Female	15 (37.3%)	14 (34.8%)		
**CGPA**			0.45	0.502
< 2	18 (45.2%)	21 (52.7%)		
≥ 2	22 (54.8%)	19 (47.3%)		
**Nationality**			1.02	0.314
Egyptian	40 (100%)	39 (97.7%)		
Non-Egyptian	0	1 (2.3%)		
**Experienced Patient Aggression**			0.21	0.648
Yes	15 (37.7%)	17 (42.7%)		
No	25 (62.3%)	23 (57.3%)		
**Fear of Psychiatric Patients**			0.20	0.653
Yes	23 (57.7%)	21 (52.7%)		
No	17 (42.3%)	19 (47.3%)		

### Psychological empowerment levels

Table 2 presents a comparative analysis of psychological empowerment levels among nursing students in the experimental and control groups before and after the implementation of the de-escalation simulation training. Before the intervention, the majority of students in both groups exhibited moderate levels of psychological empowerment, with 60.2% of the experimental group and 57.7% of the control group falling into this category. A smaller proportion of participants reported high empowerment levels pre-training, accounting for 15.2% in the experimental group and 12.2% in the control group. Notably, a substantial portion of both groups also reported poor empowerment at baseline (25.2% and 30.2%, respectively).

Following the training, there was a marked shift in the distribution of empowerment levels among the experimental group. The proportion of students classified as highly empowered increased significantly to 87.3%, while none remained in the poor empowerment category. Additionally, those in the moderate category dropped sharply to 12.7%, indicating a positive migration to higher empowerment levels. In contrast, the control group showed minimal change, with 52.7% remaining at moderate levels and only a slight increase in the proportion of students achieving high empowerment (from 12.2% to 19.7%). Alarmingly, 27.7% of control group students continued to report poor empowerment levels post-intervention. The chi-square test revealed a statistically significant difference between groups post-training (χ² = 21.73, *p* < 0.001), underscoring the effectiveness of the simulation-based intervention in enhancing psychological empowerment among nursing students.

**Table 2:** Psychological empowerment levels pre and Post-Training (*n* = 80)

Psychological empowerment	Experimental (*n* = 40) Pre *N* (%)	Experimental (*n* = 40) Post *N* (%)	Control (*n* = 40) Pre *N* (%)	Control (*n* = 40) Post *N* (%)	χ²	*P*-value
Poor	10 (25.2%)	0	12 (30.2%)	11 (27.7%)	21.73	< 0.001*
Moderate	24 (60.2%)	5 (12.7%)	23 (57.7%)	21 (52.7%)		
High	6 (15.2%)	35 (87.3%)	5 (12.2%)	8 (19.7%)		

### Detailed psychological empowerment dimensions

Table 3 presents a comparative analysis of the post-training psychological empowerment dimensions between the experimental and control groups. The data reveal that the experimental group, who received the de-escalation simulation training, achieved significantly higher mean scores across all four dimensions of empowerment—meaning, competence, self-determination, and impact—compared to their counterparts in the control group. Specifically, the mean score for the *meaning* dimension was 18.52 (± 2.73) in the experimental group versus 16.42 (± 4.58) in the control group, indicating a statistically significant difference (t = 2.43, *p* = 0.017), suggesting that students who participated in the simulation perceived their roles as more purposeful and personally valuable. The *competence* dimension, reflecting the students’ belief in their capabilities to perform effectively, was markedly higher among the trained group (18.77 ± 2.09) compared to the control group (15.82 ± 4.38), with a highly significant difference (t = 3.81, *p* < 0.001). Similar trends were observed in *self-determination* and *impact* dimensions, where the experimental group scored 17.92 (± 2.90) and 17.77 (± 3.00) respectively, as opposed to 14.72 (± 4.20) and 14.57 (± 4.30) in the control group (*p* < 0.001 for both dimensions).

**Table 3:** Post-Training psychological empowerment dimensions (*n* = 80)

Dimensions	Experimental mean ± SD	Control mean ± SD	t-value	*P*-value
Meaning	18.52 ± 2.73	16.42 ± 4.58	2.43	0.017*
Competence	18.77 ± 2.09	15.82 ± 4.38	3.81	< 0.001*
Self-determination	17.92 ± 2.90	14.72 ± 4.20	3.88	< 0.001*
Impact	17.77 ± 3.00	14.57 ± 4.30	3.89	< 0.001*

### Clinical confidence in managing patient aggression

Table 4 presents the distribution of clinical confidence levels in coping with patient aggression among nursing students in both experimental and control groups before and after the intervention. Prior to the training, the majority of participants in both groups reported low confidence—87.7% in the experimental group and 85.2% in the control group—indicating a comparable baseline. However, after the de-escalation simulation training, a substantial shift occurred in the experimental group, where only 5.2% remained in the low confidence category, and the proportion of students with high confidence rose sharply to 59.7%. In contrast, the control group exhibited a more modest improvement, with 55.2% still reporting low confidence and only 9.6% achieving high confidence post-training. The proportion of students with moderate confidence increased equally in both groups to 35.2%, suggesting some benefit from routine training. The chi-square test yielded a value of 36.48 and a highly significant p-value of < 0.001, confirming that the observed improvements in the experimental group were statistically significant and attributable to the simulation-based intervention.

**Table 4:** Clinical confidence levels pre and Post-Training (*n* = 80)

Clinical confidence	Experimental pre *N* (%)	Experimental post *N* (%)	Control pre *N* (%)	Control post *N* (%)	χ²	*P*-value
Low	35 (87.7%)	2 (5.2%)	34 (85.2%)	22 (55.2%)	36.48	< 0.001*
Moderate	5 (12.3%)	14 (35.2%)	6 (14.7%)	14 (35.2%)		
High	0	24 (59.7%)	0	4 (9.6%)		

### Correlation between empowerment and clinical confidence

Table 5 presents the correlation between psychological empowerment and clinical confidence in coping with patient aggression among nursing students in both the experimental and control groups, measured before and after the intervention. In the experimental group, a moderate positive correlation was found pre-training (*r* = 0.38, *p* = 0.045), which slightly decreased but remained statistically significant post-training (*r* = 0.24, *p* = 0.017). This suggests that students who reported higher levels of empowerment also tended to express greater confidence in managing patient aggression, and this relationship persisted even after the de-escalation simulation training. For the control group, the correlation was weak at both time points but statistically significant (pre-training: *r* = 0.01, *p* = 0.043; post-training: *r* = 0.19, *p* = 0.037), indicating a subtle yet consistent link between the two variables. These findings support the notion that while empowerment and confidence are interrelated constructs, the strength of their association is more pronounced when students undergo structured simulation-based training.

**Table 5:** Correlation between empowerment and confidence (*n* = 80)

Group	Pre-training (*r*, *P*-value)	Post-training (*r*, *P*-value)
Experimental	0.38 (0.045*)	0.24 (0.017*)
Control	0.01 (0.043*)	0.19 (0.037*)

### Comparison of total scores (empowerment & confidence)

Table 6 presents a comparative analysis of the total psychological empowerment and clinical confidence scores between the experimental and control groups following the de-escalation simulation training. The data reveal a statistically significant improvement in both variables among students in the experimental group compared to those in the control group. Specifically, the mean score for psychological empowerment in the experimental group was 72.98 (± 8.12), markedly higher than the control group’s mean of 61.52 (± 11.57), with a t-value of 5.00 and a highly significant p-value (< 0.001). This suggests that the simulation training effectively fostered a stronger sense of autonomy, competence, and influence in clinical contexts. Likewise, the experimental group demonstrated a significantly elevated mean score in clinical confidence (88.37 ± 9.22) compared to the control group (64.75 ± 12.45), with a t-value of 9.39 and an equally significant p-value (< 0.001).

**Table 6:** Comparison of total scores Post-Training (*n* = 80)

Variables	Experimental mean ± SD	Control mean ± SD	t-value	*P*-value
Total Empowerment	72.98 ± 8.12	61.52 ± 11.57	5.00	< 0.001*
Total Clinical Confidence	88.37 ± 9.22	64.75 ± 12.45	9.39	< 0.001*

## Discussion

This study investigated the impact of a de-escalation standardized patient simulation training on psychological empowerment and confidence in coping with patient aggression among psychiatric nursing students. The findings demonstrated a significant improvement in both outcomes among students who underwent the simulation training compared to those who received traditional instruction. These results underscore the importance of incorporating simulation-based education into psychiatric nursing curricula to enhance students’ clinical readiness and psychological resilience.

Psychiatric and mental health nursing is inherently challenging, especially for students who are often unprepared to manage the complexities of aggressive patient behaviors [27]. The pre-intervention findings of this study revealed that most participants exhibited low confidence and moderate to low levels of empowerment—findings that are consistent with prior literature indicating that nursing students frequently enter psychiatric settings with high levels of anxiety and low self-efficacy [28, 29]. This may be attributed to stigma, lack of clinical exposure, and fear of unpredictable behavior, which have been shown to negatively affect therapeutic communication and learning [30, 31].

Following the intervention, students in the experimental group exhibited substantial gains in both psychological empowerment and clinical confidence, with statistically significant improvements in all four empowerment domains—meaning, competence, self-determination, and impact. This aligns with previous studies that found simulation training enhances self-perceived control, competence, and autonomy in clinical decision-making [32]. The structured nature of simulation, involving realistic scenarios and immediate feedback, likely facilitated reflective learning and deepened understanding of professional roles [33].

The positive effect of simulation training on empowerment observed in this study may also be understood through the lens of Bandura’s social cognitive theory, which posits that mastery experiences—such as successful de-escalation in a simulated environment—are key to developing self-efficacy and empowered behavior [34]. By practicing verbal and non-verbal strategies in a risk-free setting, students are better able to internalize these skills and apply them with confidence in real-life situations [35]. This reinforces the importance of experiential learning in bridging the theory–practice gap that has long been criticized in nursing education [36].

The significant rise in clinical confidence scores post-intervention further supports the value of simulation in preparing students for psychiatric care. Similar studies have demonstrated that simulation improves students’ perceived preparedness, communication skills, and ability to respond to patient aggression effectively [36]. For example, Mosher (2021) reported that virtual de-escalation scenarios enhanced self-confidence among nursing students when coping with violent encounters [37]. Likewise, a quasi-experimental study in Taiwan found that simulation-based training reduced anxiety and increased aggression management confidence among novice nurses [38].

Another key contribution of this study is the observed positive correlation between empowerment and clinical confidence, both before and after the intervention. This association is well-documented in literature, as empowerment fosters a sense of agency and decision-making capacity, which are crucial for confident clinical behavior [39]. Empowered students are more likely to act decisively, maintain emotional regulation, and establish therapeutic alliances even under pressure [40]. Therefore, fostering psychological empowerment should be seen as a core objective of psychiatric nursing education, not merely an ancillary benefit.

Furthermore, the findings of this study support international efforts to reform nursing education by integrating non-technical skills such as communication, emotional intelligence, and conflict resolution into training frameworks [41]. De-escalation is increasingly recognized as an essential skill for mental health professionals, replacing outdated coercive interventions such as restraints and seclusion, which carry ethical and legal concerns [15]. Educators must thus prioritize strategies that promote safety through therapeutic interaction, empathy, and shared problem-solving—skills that were emphasized in this study’s simulation modules.

The observed reduction in the correlation between empowerment and confidence after the intervention warrants reflection. While both constructs increased in absolute terms, the weaker association may reflect several mechanisms. First, ceiling effects in confidence scores among the intervention group likely compressed variability, attenuating the strength of the relationship. Second, simulation-based practice may preferentially boost task-specific self-efficacy—that is, confidence in enacting concrete de-escalation micro-skills—more rapidly than broader psychological empowerment, which encompasses meaning, self-determination, and perceived impact. This creates a short-term decoupling between the constructs. Third, empowerment may require longer consolidation through repeated clinical exposure and opportunities for autonomous decision-making, while confidence often responds quickly to structured rehearsal. These findings highlight the importance of aligning simulation-based de-escalation training with curricular strategies that explicitly cultivate empowerment (e.g., leadership roles, reflective practice, shared decision-making exercises). They also suggest the need for longitudinal follow-up to determine whether empowerment and confidence realign over time once initial gains stabilize and transfer to clinical practice.

Our findings reinforce simulation as an effective strategy for preparing nursing students to manage aggression, with demonstrable gains in empowerment and confidence. Beyond statistical improvements, the structured design of this program—detailing session objectives, standardized patient scripts, fidelity checks, and structured debriefing—makes it highly replicable in diverse nursing curricula. By documenting the control curriculum, mapping scenario content to specific micro-skills, and embedding quality assurance through standardized tools, this study provides a transparent blueprint for educators seeking to adopt or adapt similar training. The practical relevance lies not only in the outcomes achieved but also in the method’s transferability to other psychiatric nursing contexts and broader healthcare education settings.

### Implications for nursing practice

The findings of this study have significant implications for psychiatric nursing education and broader clinical training. Incorporating simulation-based de-escalation training into undergraduate nursing curricula equips students with essential communication and crisis intervention skills that cannot be developed through didactic learning alone. As students encounter increasingly complex patient behaviors, particularly in psychiatric and emergency settings, simulation provides a safe and structured platform to develop confidence, competence, and psychological readiness. Furthermore, the observed positive correlation between empowerment and clinical confidence emphasizes the need to foster both cognitive and affective domains of learning in clinical education. Nurse educators, academic institutions, and regulatory bodies should consider adopting standardized patient scenarios focused on aggression management as a core component of pre-licensure training. Such educational investments are likely to yield a more competent, emotionally resilient, and professionally empowered nursing workforce, capable of maintaining therapeutic relationships and ensuring safety in volatile clinical environments.

### Limitations of the study

Although this study demonstrated significant and meaningful improvements in empowerment and confidence, several limitations must be acknowledged. First, the reliance on self-report questionnaires introduces the possibility of response and social desirability bias, as students may have provided answers that reflect perceived expectations rather than their actual experiences. Second, the study only measured immediate post-intervention outcomes and did not include a longitudinal follow-up. As a result, it remains unclear whether the observed gains in empowerment and confidence would persist over time or translate into clinical practice. Third, the study employed a convenience sample drawn from a single academic institution, which limits the generalizability of findings to other nursing programs or cultural contexts. Finally, while the standardized patient simulation offered a structured and safe learning environment, it cannot fully replicate the unpredictability, emotional intensity, and contextual complexity of real-world aggressive encounters in psychiatric settings. Future research should address these limitations by employing multi-site designs, objective behavioral assessments, and long-term evaluations of simulation training effectiveness.

## Conclusion

This randomized controlled trial demonstrates that de-escalation simulation training significantly enhances psychiatric nursing students’ empowerment and confidence in coping with patient aggression. The inclusion of detailed scenario maps, curriculum comparisons, and fidelity measures ensures that the intervention is transparent, reproducible, and adaptable to varied institutional contexts. These features extend the study’s impact beyond immediate outcomes, offering educators a clear, evidence-based model for strengthening aggression management training. Future research should examine the long-term sustainability of these gains, their transfer to real clinical encounters, and integration with broader empowerment-oriented strategies in nursing education.

## Supplementary Information

Below is the link to the electronic supplementary material.

Supplementary Material 1

Supplementary Material 2

## Data Availability

The datasets used and/or analyzed during the current study are available from the corresponding author on reasonable request.
