# Supplementary material for: The effect of de-escalation simulation training on empowerment and confidence in managing patient aggression among psychiatric nursing students: an experiential learning approach
Source: BMC Nurs. 2026 Jan 12;25:26. doi: 10.1186/s12912-025-03958-1 (PMC12797913; doi:10.1186/s12912-025-03958-1)
Supplement: Supplementary file 1 — Supplementary Material 1 [file 12912_2025_3958_MOESM1_ESM.docx]

# Supplementary File S1

## Simulation Training Scenarios and Structure

### Overview

The experimental group participated in a structured de-escalation simulation program consisting of 12 sessions (4 theoretical, 8 practical) delivered over eight weeks. The training was designed in alignment with the International Nursing Association for Clinical Simulation and Learning (INACSL) Standards of Best Practice, Bandura’s self-efficacy framework, and Hameli’s psychological empowerment model.

### Theoretical Sessions

- Introduction to aggression in psychiatric settings (definitions, prevalence, impact on staff and patients).
- Principles of de-escalation: verbal and non-verbal techniques.
- Communication strategies: active listening, empathy, maintaining therapeutic boundaries.
- Legal and ethical aspects of managing aggression.

### Practical Simulation Sessions

Each simulation was conducted with trained standardized patients (SPs) portraying escalating aggression. Sessions lasted 10–15 minutes, followed by structured debriefing.

Example Scenarios:

1. Verbal Threats: A patient expressing anger over delayed medication, using hostile language and body posture.

2. Refusal of Medication: A patient loudly refusing treatment, displaying agitation and pacing the room.

3. Escalating Agitation: A patient with hallucinations becoming increasingly suspicious and defensive, raising their voice and gesturing aggressively.

4. Boundary Testing: A patient repeatedly ignoring instructions, intruding on personal space, and displaying intimidating behavior.

### Debriefing Process

After each simulation, students engaged in guided reflection covering:
- Emotional responses to aggression.
- Evaluation of communication strategies used.
- Strengths and areas for improvement.
- Reinforcement of empowerment and confidence in clinical practice.

### Control Group

The control group continued with routine teaching methods, including lectures, textbook readings, and class discussions. No simulation, role-play, or SP-based practice was provided.
