# Supplementary material for: The effect of de-escalation simulation training on empowerment and confidence in managing patient aggression among psychiatric nursing students: an experiential learning approach
Source: BMC Nurs. 2026 Jan 12;25:26. doi: 10.1186/s12912-025-03958-1 (PMC12797913; doi:10.1186/s12912-025-03958-1)
Supplement: Supplementary file 2 — Supplementary Material 2 [file 12912_2025_3958_MOESM2_ESM.docx]

# Supplementary File S2

## Data Collection Instruments

### 1. Psychological Empowerment Scale (PES)

Original Developer: Spreitzer (1995)
Adaptation/Validation: Ruiz-Fernández et al. (2022); Arabic translation and validation conducted for the current study.

Aim: To measure psychological empowerment across four domains.

Structure:
- 12 items, divided into four subscales:
 • Meaning (3 items)
 • Competence (3 items)
 • Self-determination (3 items)
 • Impact (3 items)

Scoring:
- 7-point Likert scale (1 = strongly disagree; 7 = strongly agree).
- Total score: 12–84.
- Interpretation: <50% = low empowerment; 50–74% = moderate; ≥75% = high.

Reliability/Validity:
- Cronbach’s alpha > 0.90 (original validation).
- For the Arabic version: translation–back translation, expert panel review (5 psychiatric nursing experts), pilot testing with 20 students; Cronbach’s alpha = 0.92.

Items (English version - examples):
1. The work I do is meaningful to me.
2. I am confident about my ability to do my job.
3. I have significant autonomy in determining how I do my job.
4. My impact on what happens in my department is large.
... (remaining items included in full version).

Arabic version: Provided in full in attached appendix (translated and validated).

### 2. Clinical Confidence in Coping with Patient Aggression Questionnaire (CCWPA)

Original Developer: Thackrey (1987)
Adaptation/Validation: Kruse (2021); Arabic translation and validation conducted for the current study.

Aim: To assess students’ perceived confidence in coping with aggressive patients.

Structure:
- 10 items.
- Responses on an 11-point Likert scale ranging from “very poor/unsafe/unable” to “very good/safe/able.”

Scoring:
- Total score: 10–110.
- Interpretation: <50% = low confidence; 50–74% = moderate; ≥75% = high.

Reliability/Validity:
- Strong internal consistency and criterion-related validity reported in previous studies.
- Arabic version validated through translation–back translation, expert review, and pilot testing; Cronbach’s alpha = 0.89.

Items (English version - examples):
1. How comfortable are you in dealing with a verbally aggressive patient?
2. How safe do you feel when confronted by escalating patient behavior?
3. How effective do you believe your de-escalation skills are?


**1. مقياس التمكين النفسي (Psychological Empowerment Scale – PES)**

**المطور الأصلي:** Spreitzer (1995)
**عدد البنود:** 12 بندًا موزعة على أربعة أبعاد (المعنى، الكفاءة، الاستقلالية، التأثير).
**مقياس الإجابة:** سلم ليكرت من 7 نقاط (1 = أعارض بشدة، 7 = أوافق بشدة).

**البنود:**

**بُعد المعنى (Meaning):**

1. العمل الذي أقوم به ذو معنى بالنسبة لي.
2. أنشطتي الدراسية/العملية ذات مغزى شخصي بالنسبة لي.
3. أشعر أن ما أتعلمه في مجال التمريض النفسي مهم للغاية.

**بُعد الكفاءة (Competence):**
4. أشعر أنني واثق من قدرتي على أداء مهامي بنجاح.
5. أمتلك المهارات اللازمة لإنجاز العمل المطلوب مني.
6. أشعر بالكفاءة العالية في التعامل مع المواقف الصعبة.

**بُعد الاستقلالية (Self-determination):**
7. أتمتع بقدر كبير من الحرية في تحديد كيفية إنجاز مهامي.
8. أستطيع اختيار الأساليب التي أستخدمها لأداء عملي.
9. أشعر أن لدي القدرة على اتخاذ قرارات مستقلة في بيئة التدريب.

**بُعد التأثير (Impact):**
10. أشعر أن ما أقوم به له تأثير واضح على نتائج العمل أو الدراسة.
11. أساهم بشكل فعّال في تحقيق أهداف القسم/الكلية.
12. أمتلك القدرة على التأثير في سير الأمور المتعلقة بالتدريب السريري.

**2. استبيان الثقة السريرية في التعامل مع عدوان المرضى**

(Clinical Confidence in Coping with Patient Aggression Questionnaire – CCWPA)

**المطور الأصلي:** Thackrey (1987)
**عدد البنود:** 10 بنود.
**مقياس الإجابة:** سلم ليكرت من 11 نقطة (0 = ضعيف جدًا/غير آمن، 10 = ممتاز جدًا/آمن جدًا).

**البنود:**

1. ما مدى ارتياحك عند التعامل مع مريض يظهر عدوانًا لفظيًا؟
2. ما مدى ثقتك في قدرتك على تهدئة مريض غاضب؟
3. إلى أي مدى تشعر أنك آمن عند مواجهة سلوك عدواني من مريض؟
4. ما مدى فعاليتك في استخدام مهارات خفض التصعيد؟
5. ما مدى ارتياحك في التواصل مع مريض يتحدى القواعد أو التعليمات؟
6. ما مدى ثقتك في قدرتك على تقييم شدة الموقف العدواني؟
7. ما مدى استعدادك للتعامل مع مريض يرفض تناول الدواء بعنف؟
8. ما مدى كفاءتك في استخدام لغة الجسد لخفض التوتر عند المريض؟
9. ما مدى ثقتك في الحفاظ على سلامتك وسلامة الآخرين أثناء الموقف العدواني؟
10. ما مدى اعتقادك بقدرتك على حل الموقف العدواني دون اللجوء إلى تدخل جسدي؟
